# Neurophysiological features of Internet gaming disorder and alcohol use disorder: a resting-state EEG study

**DOI:** 10.1038/tp.2015.124

**Published:** 2015-09-01

**Authors:** K-L Son, J-S Choi, J Lee, S M Park, J-A Lim, J Y Lee, S N Kim, S Oh, D J Kim, J S Kwon

**Affiliations:** 1 Department of Neuropsychiatry, Seoul National University Hospital, Seoul, Republic of Korea; 2 Department of Psychiatry, SMG-SNU Boramae Medical Center, Seoul, Republic of Korea; 3 Department of Psychiatry and Behavioral Science, Seoul National University College of Medicine, Seoul, Republic of Korea; 4 Department of Psychiatry, Gangnam Eulji Hospital, Eulji University, Seoul, Republic of Korea; 5 Department of Biostatistics, SMG-SNU Boramae Medical Center, Seoul, Republic of Korea; 6 Department of Psychiatry, Seoul St Mary's Hospital, The Catholic University of Korea College of Medicine, Seoul, Republic of Korea

## Abstract

Despite that Internet gaming disorder (IGD) shares clinical, neuropsychological and personality characteristics with alcohol use disorder (AUD), little is known about the resting-state quantitative electroencephalography (QEEG) patterns associated with IGD and AUD. Therefore, this study compared the QEEG patterns in patients with IGD with those in patients with AUD to identify unique neurophysiological characteristics that can be used as biomarkers of IGD. A total of 76 subjects (34 with IGD, 17 with AUD and 25 healthy controls) participated in this study. Resting-state, eyes-closed QEEGs were recorded, and the absolute and relative power of brains were analyzed. The generalized estimating equation showed that the IGD group had lower absolute beta power than AUD (estimate=5.319, *P*<0.01) and the healthy control group (estimate=2.612, *P*=0.01). The AUD group showed higher absolute delta power than IGD (estimate=7.516, *P*<0.01) and the healthy control group (estimate=7.179, *P*<0.01). We found no significant correlations between the severity of IGD and QEEG activities in patients with IGD. The current findings suggest that lower absolute beta power can be used as a potential trait marker of IGD. Higher absolute power in the delta band may be a susceptibility marker for AUD. This study clarifies the unique characteristics of IGD as a behavioral addiction, which is distinct from AUD, by providing neurophysiological evidence.

## Introduction

Internet gaming disorder (IGD) is defined as the repetitive use of Internet-based games leading to significant difficulties with functioning.^1^ It is well known that IGD is related to various comorbid psychological conditions, such as depressed mood^2^ and anxiety,^3^ as well as psychiatric disorders, including attention deficit hyperactivity disorder^4^ and obsessive-compulsive disorder.^5^ Many researchers have classified IGD as a behavioral addiction, a category that includes pathological gambling and sexual activity.^6, 7^ These disorders share clinical features, such as compulsive behavior, lack of control over the problematic behavior and a craving state before engagement in the problematic behavior.^8^ IGD has also been conceptualized as an impulse control disorder.^9, 10^ A study of Internet addiction among Chinese adolescents found a correlation between Internet addiction and impulsivity.^11^ Lee *et al.*^12^ compared the impulsivity of patients with IGD with that of those with pathological gambling, and found that patients with IGD showed higher levels of trait impulsivity than did those with pathological gambling. In addition, patients with IGD showed similar impulsivity patterns to those with alcohol use disorder (AUD) in terms of trait impulsivity and stop-signal test.^13, 14^ The American Psychiatric Association subsumed IGD in the behavioral addictions category in the draft of the diagnostic criteria for *Diagnostic and Statistical Manual of Mental Disorders, Fifth Edition* (DSM-5) released in 2010. However, IGD was ultimately included in an appendix to DSM-5 because of insufficient evidence. It is therefore of interest to compare IGD with AUD, one of substance use disorders, to investigate the pathophysiology of IGD and to clarify its characteristics as an addictive disorder.

Spontaneous brain activity under the resting-state, eyes-closed condition has been increasingly identified as the brain activity correlate of cognition and behavior.^15^ The resting-state electroencephalography (EEG) represents the ongoing oscillation of brain electrical activity that occurs while the person being examined is relaxing.^16^ A brain region known as the default mode network increases its activity during the resting state, which reflects spontaneous cognitive processes.^17^ Resting-state brain activity is also associated with the event-related cognitive processes involved in attention, memory and thinking.^18^ Therefore, analysis of resting-state EEGs should help us to understand the differences and similarities in the basic brain function of patients with IGD versus those with AUD.

Several studies on the resting-state EEGs of individuals with IGD have been conducted. Patients with IGD showed lower absolute beta power in the resting state, and this was correlated with the severity of impulsivity.^19^ Lee *et al.*^20^ examined the difference in the resting-state EEG activity of patients with Internet addiction and comorbid depression compared with patients with Internet addiction without depression. Their results indicated that patients with Internet addiction without depression showed lower absolute delta and beta power compared with patients with Internet addiction with depression, suggesting lower absolute delta and beta power as potential biomarkers of Internet addiction.

Studies regarding the resting-state EEGs of individuals with AUD have consistently reported that the EEG activities of these patients showed higher absolute beta power than did those of healthy controls. Rangaswamy *et al.*^21^ reported that the beta power of the resting-state EEGs was elevated in those with AUD, and suggested that this may be an electrophysiological index of an imbalance in the excitation–inhibition homeostasis in the cortex. Coutin-Churchman *et al.*^22^ also reported that higher beta activity is one of the most frequent quantitative EEG (QEEG) alterations in patients with alcoholism, and that this QEEG activity significantly correlated with clinical severity, suggesting that this feature may be a marker of cortical hyperexcitability.

As mentioned above, it is of interest to compare IGD with AUD to investigate the pathophysiology of IGD and to clarify its neurobiological characteristics as an addictive disorder. However, no direct comparisons of the neurophysiological features of IGD and AUD have been conducted to date. The purpose of the present study was to investigate the neurophysiological similarities or differences of IGD with AUD and healthy controls by examining the resting-state QEEG among treatment-seeking patients with IGD or AUD, and healthy controls. In addition, unlikely with patients with AUD, whose neurophysiological features can be influenced by substances, the QEEG features of patients with IGD can show unique neurophysiological features of addiction, not contaminated by substances. Therefore, the present study can also provide evidence of intrinsic QEEG features of behavioral addiction compared with those of healthy controls and substance use disorder. As we stated above, we hypothesized that, compared with healthy controls, patients with IGD would show lower absolute beta power, whereas patients with AUD would show higher absolute beta power.

## Materials and methods

### Participants

We recruited a total of 76 young males to participate in this study: 34 were diagnosed with IGD (age: 22.71±5.47 years), 17 were diagnosed with AUD (age: 29.71±4.88 years) and 25 were healthy controls (age: 23.88±4.66 years). All patients were seeking treatment at the outpatient clinics of SMG-SNU Boramae Medical Center in Seoul, Republic of Korea due to excessive participation in Internet gaming or alcohol consumption.

Patients with IGD were diagnosed according to DSM-5 criteria, and Young's Internet Addiction Test^10^ was used to assess the severity of participants' IGD. Previous studies have defined excessive Internet users as those with Internet Addiction Test total scores of at least 50.^10, 23^ We included those subjects with Internet Addiction Test scores of at least 70 who spent more than 4 h per day and 30 h per week using Internet games to study only those with severe IGD rather than those who were merely at high risk for developing this disorder due to excessive Internet gaming. The mean Internet Addiction Test score of patients in the IGD group was 75.09±5.81, and their mean times spent using Internet games per weekday and per weekend day were 6.06±2.78 and 7.79±2.77 h, respectively. In addition, the Structured Clinical Interview for DSM-IV was used to identify past and current psychiatric illnesses.

Diagnoses of AUD were based on DSM-5 criteria and were made by a clinically experienced psychiatrist. The severity of AUD was assessed by the Korean version of Alcohol Use Disorder Identification Test-Korea (AUDIT-K).^24^ The mean AUDIT-K score for the AUD group was 25.71±5.37, and the mean amount of alcohol consumed per day by this group was 10.35±2.89 standard drinks. Patients with AUD used Internet games <2 h per day and had abstained from alcohol use for at least 2 weeks before participation in the study. Abstinence from alcohol was verified by self-reports and reports from caregivers. We regarded these reports as reliable because the participants attended regular follow-up visits to our outpatient clinic and showed good adherence to treatment.

Healthy controls were recruited from the local community and had no history of any psychiatric disorder. Healthy controls played Internet games <2 h per day and drank fewer than 14 standard drinks per week and fewer than four standard drinks per occasion. They also had no lifetime history of AUD.

Participants also completed the Beck Depression Inventory (BDI),^25^ the Beck Anxiety Inventory (BAI)^26^ and the Barratt Impulsiveness Scale version 11 (BIS-11).^27^ The BDI and the BAI were administered to all subjects to measure depressive and anxiety symptoms, respectively. The BIS-11 was used to measure trait impulsivity. All scales had been validated in Korea. Exclusion criteria were a history of significant head injury, seizure disorder, mental retardation and psychotic disorder. All participants were medication naive at the time of assessment. The Korean version of the Wechsler Adult Intelligence Scale-III was administered to all subjects to estimate intelligence quotient, and we included only subjects with Wechsler Adult Intelligence Scale-III scores of at least 80. The Institutional Review Board of the SMG-SNU Boramae Medical Center approved the study protocol, and all subjects provided written informed consent before participation.

### EEG recording

Detailed descriptions of EEG recordings and data acquisition were presented in our previous report.^19^ The participants were seated and engaged in a resting state in an isolated sound-shielded room connected to a recording room via a one-way glass window. EEG recordings lasted for 10 min and included the following conditions: 4 min with eyes closed, 2 min with eyes open and 4 min with eyes closed.

EEG recordings and acquisitions were made using SynAmps2 (Compumedics, Abbotsford, VIC, Australia) with a 64-channel Quik-cap and a NeuroScan system (Scan 4.3; Compumedics). A reference, single channel with bipolar electrodes, was attached to the mastoids. The location of the ground channel was between FPz and Fz. The signals were sampled at a frequency of 500 Hz. The electrode impedance was below 5 kΩ, and the EEG signal was band-pass filtered at 0.1–60 Hz using Scan 4.3. Recordings from the NeuroScan system were transferred to NeuroGuide software (NG 2.5.5; Applied Neuroscience, St Petersburg, FL, USA) for spectral analysis in a 32-bit file format, and 19 sites of 64 channels were driven by the following montage set of NeuroGuide: FP1, F3, F7, Fz, FP2, F4, F8, T3, C3, Cz, T4, C4, T5, P3, O1, Pz, T6, P4 and O2. Artifact removal was performed offline using the artifact rejection toolbox of NeuroGuide software. EEG recordings were also visually inspected to eliminate eye muscle movements and other artifacts, and artifact-free epochs of 20–60 s under eyes-closed conditions were selected for spectral analysis. Accepted epochs of EEG data for both absolute (uV^2^) and relative (%) power were smoothed using fast Fourier transforms and averaged in four frequency bands by NeuroGuide's spectral analysis system: delta (0.5–4 Hz), theta (4–8 Hz), alpha (8–12 Hz) and beta (12–30 Hz). Source localization and visualization based on the 10/20 system was performed with Matlab software (MathWorks, Natick, MA, USA). In addition, the activity at 19 sites was divided into three regions by averaging within each region: frontal (FP1, F3, F7, Fz, FP2, F4 and F8), central (T3, C3, Cz, T4 and C4) and posterior (T5, P3, O1, Pz, T6, P4 and O2).

### Statistical analysis

Before the formal analysis, we conducted an exploratory data analysis to identify and remove outliers to avoid the possibility of spurious results. As repeated or multiple outcomes from the same subject are correlated, the statistical methods that are affected by those correlations among outcomes should be considered. In this study, a generalized estimating equation (GEE),^28^ which is an extension of the generalized linear model for multivariate responses, was used to assess the group effect on the absolute or relative power in each band. The GEE has been used to analyze the characteristics of EEGs.^29, 30, 31, 32, 33, 34, 35^ In this study, group (IGD versus AUD versus healthy control), region and their interaction effects were tested using GEE. The group-by-region interaction term was used to detect differential group effects in the absolute or relative power in each band by region. In the absence of evidence of an interaction effect, the effect of group was tested. In addition, we adjusted for the effect of age because of differences in the ages between patients with IGD and those with AUD.

Comparisons of demographic and clinical variables among groups were performed using analysis of variance. We used Pearson's correlations to explore correlations between EEG activities and demographic/clinical variables in patients with IGD, or AUD, and healthy controls. Statistical analyses were performed using IBM SPSS Statistics version 20 (IBM, Armonk, NY, USA) and R version 2.15.2 (http://www.r-project.org), and *P*-values <0.05 were considered statistically significant. In addition, for the correlational analyses, *P*-value <0.01, adjusted for multiple corrections, was set as significant.

## Results

### Demographic and clinical data

The demographic and clinical characteristics of participants are presented in Table 1. We found no significant differences in education or intelligence quotient among the groups. Patients with AUD were older than were those with IGD. However, there were no significant differences in age between patients with AUD and healthy controls or between patients with IGD and healthy controls. Both the IGD and AUD groups had higher BDI (*P*<0.05), BAI (*P*<0.05) and BIS-11 (*P*<0.05) scores than did controls.

### EEG activity

#### Absolute power

**Figure 1:**
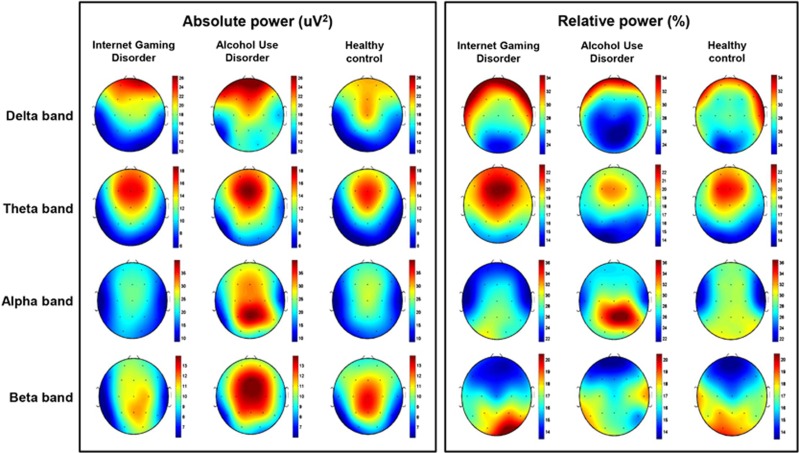
Topographical maps of absolute and relative power in the Internet gaming disorder (IGD), alcohol use disorder (AUD) and healthy control groups. Scales show uV^2^ for absolute power and % for relative power. Red represents higher values and blue represents lower values. The IGD group demonstrated reduced absolute power in the beta band, compared with the AUD and healthy control groups. The AUD group demonstrated higher absolute power in the delta band compared with the IGD and healthy control groups. We found no significant main group effect in the relative power for all bands.

**Figure 2:**
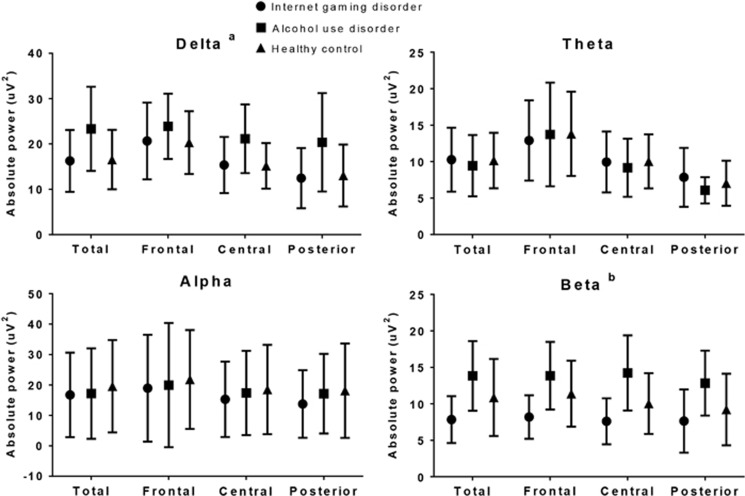
Absolute power in each band under the resting-state eyes-closed condition. The horizontal bars represent s.d. (**a**) Absolute delta power: alcohol use disorder > Internet gaming disorder=healthy controls (*post hoc*, *P*<0.05). (**b**) Absolute beta power: alcohol use disorder=healthy control > Internet gaming disorder (*post hoc*, *P*<0.05).

Figure 1 shows the scalp topography of the three groups in terms of the absolute power in each band. The GEE results showed no significant group-by-region interaction effect for the absolute power in each band. However, we found a main group effect in the absolute power in the delta band (*P*<0.01). The AUD group showed higher absolute power in the delta band than did the IGD (estimate=7.516, *χ*^2^=17.539, *P*<0.01) and the healthy control (estimate=7.179, *χ*^2^=17.539, *P*<0.01) groups. In addition, we found a main group effect in the absolute power in the beta band (*P*<0.01). The IGD group showed lower absolute power in the beta band than did the AUD (estimate=5.319, *χ*^2^=17.539, *P*<0.01) and the healthy control (estimate=2.612, *χ*^2^=17.539, *P*=0.01) groups (Figure 2).

With respect to theta and alpha bands, we found no group-by-region interaction effect for absolute power, and the main group effect was not significant in any of three regions.

#### Relative power

**Figure 3:**
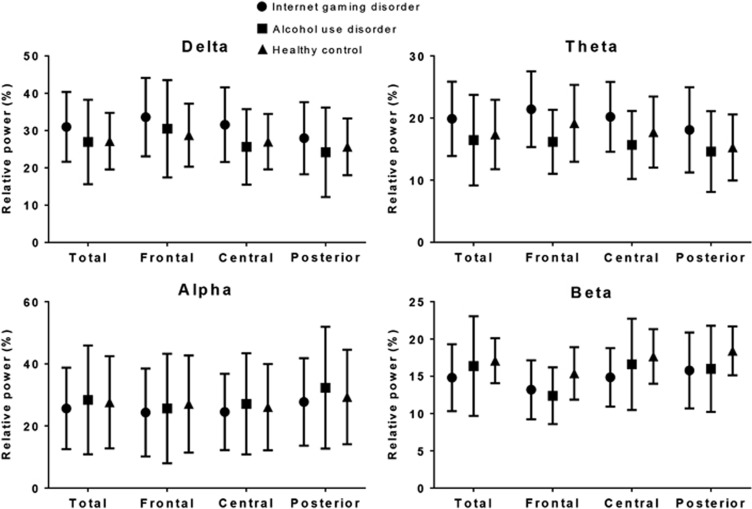
Relative power in each band under the resting-state eyes-closed condition. The horizontal bars represent s.d.

Figure 1 shows the scalp topography of the three groups for the relative power in each band. The GEE results revealed no group-by-region interaction effect for the relative power in each band. The main group effect was not significant for any of the bands in any of the three regions (Figure 3).

#### Correlations between clinical variables and EEG activity

We found no significant correlations between clinical variables and EEG powers in each band among either patients with IGD or those with AUD.

## Discussion

The present study investigated similarities or differences in the eyes-closed resting-state QEEGs of patients with IGD or AUD and healthy controls. As hypothesized, patients with IGD showed lower absolute beta power compared with patients with AUD and healthy controls. A previous study suggested that lower absolute beta power in the resting state can be a possible neurobiological trait marker of IGD versus healthy controls.^19^ The findings from the present study, significant difference in absolute beta power levels comparing IGD group with AUD group and healthy controls, provides evidence supporting the potential use of absolute beta power as a trait biomarker, which is consistent with findings from previous literature.^19^ Lee *et al.*^20^ also reported that pure IGD patients without depression showed lower absolute beta and delta powers compared with both IGD patients with depression and healthy controls. In the present study, patients with IGD showed lower absolute delta power compared with patients with AUD (IGD versus AUD: estimate=-7.516, *χ*^2^=17.539, *P*=0.001). In addition, although not statistically significant, patients with IGD showed lower absolute delta power compared with healthy controls (IGD versus healthy control: estimate=-0.337, *χ*^2^=17.539, *P*=0.838). Thus, it is possible that absolute delta power as well as absolute beta power can be neurophysiological feature that can distinguish IGD from AUD or healthy controls. In addition, building on previous studies reported increased ratios of theta/beta and delta/beta waves for gambling disorder,^36, 37^ the present study could provide unique neurophysiological evidence that patients with IGD showed lower absolute beta power, which was not followed by higher absolute delta and theta powers, thereby suggesting that lower absolute beta power without higher absolute delta and theta powers could be a candidate neurophysiological biomarker of IGD distinguishing from other behavioral addictions such as gambling disorder. In this study, we could not investigate the difference between IGD and other behavioral addictions such as gambling disorder. Further investigation that can provide evidence of similarities or differences between IGD and other behavioral addictions is needed.

Patients with AUD showed higher absolute delta power than did patients with IGD and healthy controls. However, we found no significant association between delta power and AUDIT-K scores among patients with AUD. This may suggest that higher absolute delta power can be treated as a potential trait biomarker of AUD, which precedes excessive alcohol consumption, rather than as a state marker, which is a consequence of alcohol consumption. Interestingly, contrary to previous findings and our hypotheses that patients with AUD showed higher beta power compared with healthy controls, we found no significant evidence of higher absolute beta power among patients with AUD compared with the other groups. Instead, the average absolute delta activity was highest in the AUD group. Previous studies regarding the resting-state EEGs of AUD patients also showed higher slow waves (delta and theta waves) in patients with AUD. After examining the resting-state eyes-closed EEGs of patients with AUD, Rangaswamy *et al.*^38^ found higher absolute theta power in alcohol-dependent subjects, particularly among males. Pollock *et al.*^39^ and Rangaswamy *et al.*^38^ reported that the slow-wave alteration was sustained even after a period of abstinence, suggesting that the higher power of slow waves could be treated as trait marker of AUD. However, many studies have also found lower slow waves in patients with AUD, which differs from the findings of the current study. Saletu-Zyhlarz *et al.*^40^ found that the EEG power of alcohol-dependent patients was characterized by decreased delta waves. Coutin-Churchman *et al.*^41^ also found lower slow (theta–delta) activity in many alcoholic patients. However, the above two studies did not measure resting-state EEG, and the mean age of participants in the study conducted by Coutin-Churchman *et al.*^41^ was much older (mean: 41.5 years, s.d.: 8.1) than the age of subjects in the current study (mean age: 29.71 years, s.d.: 4.88). Therefore, the discrepancies in the results regarding the absolute delta waves of patients with AUD may be due to differences in measurement and study populations.

In the present study, we found more differences than commonalities in the neurophysiological activities of patients with IGD and those with AUD. As expected, we found significantly lower average absolute beta power during the resting state among patients with IGD than in those with AUD or healthy controls. This may represent a unique pattern of resting-state EEG among patients with IGD, as a behavioral addiction, compared with patients with AUD, as a substance use disorder.

This study has several limitations. First, the sample size was insufficient to represent all patients with IGD and AUD. In addition, we investigated only male subjects. Therefore, the generalizability of results of the present study may be limited. Second, we found significant differences in depressive (BDI) and anxiety (BAI) symptoms between the IGD and AUD groups. Both the BDI and BAI scores were significantly higher in the AUD group than in the IGD group. In terms of the potential bias due to the differential distribution of BDI and BAI scores between the groups, we performed sensitivity analyses by including BDI and BAI in the GEE models. We observed no notable difference in the point estimate of the IGD group versus the AUD group with regard to the absolute delta and beta power values before and after including the two variables. Further investigations with a larger study population and more variables that potentially influence the resting-state QEEG are necessary.

This study has several strengths. First, all participants enrolled in this study were medication naive. Because QEEG activity can be influenced by medications, such as antidepressants^42^ and benzodiazepines,^43^ our inclusion of only medication-naive participants was able to eliminate the potential bias due to medication. In addition, we believe this is the first study to compare resting-state EEG activity among young patients with IGD, or AUD, and healthy controls. Almost all previous studies conducted in patients with AUD included subjects who were older than those in the current study. Although patients with AUD were older than those with IGD, we made a considerable effort to recruit young adults with AUD to allow for meaningful comparisons with the young subjects with IGD.

In summary, our results showed that IGD was distinguishable from AUD as an addictive disorder in that the resting-state EEG activity of those with IGD was lower in absolute beta power. This difference in resting-state EEG activity may be a neurobiological marker for the IGD, with lower absolute beta power as a trait marker. This study clarifies the unique characteristics of IGD as a behavioral addiction, which is distinct from AUD, by providing neurophysiological evidence.

## Figures and Tables

**Table 1:** Demographic and clinical characteristics in study subjects

	*Internet gaming disorder (*N=*34)*		*Alcohol use disorder (*N=*17)*		*Healthy controls (*N=*25)*		F	P	Post hoc
*Demographic data*									
Age (years)	22.71	5.47	29.71	4.88	23.88	4.66	11.17	<0.01	A>I=H
Education (years)	13.09	2.42	13.88	2.47	14.44	2.06	2.51	0.09	
IQ score	114.06	17.21	110.88	10.07	120.24	9.78	2.65	0.08	
									
*Clinical data*									
Duration of illness (years)	7.32	3.68	5.24	2.63	—	—	4.34	0.04	I>A
IAT	75.09	5.81	28.53	5.79	26.2	8.92	443.54	<0.01	I>A=H
AUDIT-K	4.2	2.03	25.71	5.37	3.08	3.21	292.1	<0.01	A>I=H
BDI	17.38	8.67	26.71	17.59	3.68	3.17	27.24	<0.01	A>I>H
BAI	15.44	8.96	24.82	18.12	6.64	4.82	14.61	<0.01	A>I>H
BIS-11	71.74	9.74	73.00	12.88	55.20	8.52	23.47	<0.01	I=A>H

Abbreviations: A, alcohol use disorder; AUDIT-K, Alcohol Use Disorder Identification Test-Korea; BAI, Beck Anxiety Inventory; BDI, Beck Depression Inventory; BIS-11, Barratt Impulsiveness Scale version 11; H, healthy control; IAT, Internet Addiction Test; I, Internet gaming disorder; IQ, intelligence quotient.

Data are presented as means (s.d.). The Bonferroni test was used for *post hoc* analyses.
